# The Surgical Treatment of Effusions into the Thorax

**Published:** 1890-08

**Authors:** Joseph Hoffman

**Affiliations:** Philadelphia, Pa., Fellow of the American Association of Obstetricians and Gynecologists; 126 West Diamond Street


### THE SURGICAL TREATMENT OF EFFUSIONS INTO THE THORAX.[1]

By JOSEPH HOFFMAN, M. D., Philadelphia, Pa.,

Fellow of the American Association of Obstetricians and Gynecologists.

In serous effusions into the thoracic cavity, we must consider ourselves on the border line, where medicines may be considered inferior to surgery or instrumental assistance, in freeing an important viscus from the burden of external pressure. The logic of early interference in abdominal effusions, where all the viscera having to do with the functions of organic growth and nutrition are subjected to harmful pressure, is so readily perceived and so generally accepted, that when I was requested by the secretary of this section to offer a paper before it, it seemed a fitting place to discuss a subject that, though generally conceded as belonging to medicine proper, more fitly pertains to surgery.

The question is all the more open to consideration from a surgical standpoint, inasmuch as when there is an effusion of whatever character of any considerable amount into the neighboring abdominal cavity, medicine is used only as an adjunct to its surgical removal, not as a primary agent. Why the dividing line of the diaphragm should justify and make orthodox such totally variant modes of treatment, is not easily, if at all, explained. Let it be understood from the beginning that the advocacy of early, or, if you please, prompt puncture in thoracic effusions, is in no wise urged as original or new. It is only sought here to discuss this treatment comparatively, with that now established to be most successful in the peritoneal cavity, in which parallel disease is frequently found, both acute and chronic.

So far as etiology is concerned, peritonitis and pleuritis frequently trace their origin to common causes. Both may originate in cold. Both have pyemia prominently to be considered in a causal relation, as are local abscesses, and nephritis and traumatism. In analogous diseases, each with so many common factors, is there a logic or plausible explanation even for such divergent methods of treatment?

But if there are so many factors common in these two effusions into the two principal vital cavities, the brain excepted, (in which it is notable that aspiration is the only recognized method of relieving fluid pressure in its ventricles,) it is to be remarked that the effect

1. Read by title at the forty-first annual meeting of the American Medical Association, in Nashville, Tenn., May, 1890.

of all these several factors, pathologically considered, is identical. They all destroy life by interfering with the vital processes in the same manner.

The same is true, of course, of all disease, but here in each there is both a physical or vital exhaustion and a mechanical exhaustion by pressure; so that we not only have a depressed vitality owing to the disease proper, but an interference with the mechanics of organic life as regulated by ordinary physical laws controlling the diffusion of gases, hydrostatic pressure, and frictional resistance. The same conclusion can be reached as to other diseases affecting these serous membranes, and the same reasoning applied to pericardial effusions. In peritonitis of puerperal origin it was formerly the custom to allow the patient to die, with her abdomen covered with poultices and filled with pus, under the idea that an existing trouble would be made much worse by interfering with it. In pleurisy there is no doubt the dread that interference by puncture may increase an inflammation already established, or convert a serous into a purulent effusion.

Such reasoning is just as fallacious in effusions of the chest as in those of the abdomen and pelvic cavity. Where there has been an inflammatory action set up, a new inflammatory process is hard to establish. In abdominal cases in which operation is done to remove pus, it is notable that the dangers of subsequent peritonitis are almost *nil*, if a clean operation is done. The patient does not *get* peritonitis, because she already had it, and interference is made to cure it.

Now, by analogy, the same state of affairs ought to obtain in the pleural cavity, and in reality it does. It has been long ago shown by the French clinicians [Castiaux, Moutard-Martin, Wedal,] that early puncture in thoracic affections is followed by no evil after-effects, but on the contrary the duration of the attack is abridged. It has also been conclusively shown that the existence of fever is no contra-indication of the operation, just as in the abdominal lesion where the peritoneum is cleansed to cure the fever. The fact that in every effusion leucocytes are present, is suggestive of a causal relation between the fluid and the fever. It is also ably argued by Formad, from an enormous post-mortem experience, that pleuritis frequently causes tuberculosis, and that even a moderate effusion after the subsidence of fever is a troublesome and dangerous foreign element, likely to yield perilous complications and results. With these pathological facts before us it would seem, in the light of the manifestly good operative results in the surgically allied affections of the peritoneal cavity, that the theory of delay is really not a logical one here, but a remnant of tradition, or perhaps a superstitious faith in the efficacy of drugs. In debilitated conditions incident to existing pleurisy, depletory medication can hardly fail to increase the exhaustion ; for salines and their analogues, after all, act on purely mechanical principles, and remove the effusion from the thorax by disarranging the hydrostatic balance, and producing an outward flux at a sacrifice of intestinal nutrition through irritant depletion. This criticism of medical depletion, of course, cannot apply where there is congestion of the portal system in hepatic disease, or to the use of pilocarpine or jaborandi, in effusions due to nephritis. Here, on the one hand, medication acts mechanically to assist an overtaxed organ, and on the other has really a curative effect by temporarily taking the strain from the congested kidneys.

Now, in addition to the mechanical effects of effusion upon the lungs, it must be remembered that the effects of compression are not limited to these organs alone. The heart suffers, and consequently the general circulation. There is venous stagnation, and diminished urinary secretion. The harmful influences at work upon the lungs are those of pressure, diminished vitality, elasticity, the production of adhesions, false membranes and of localized pneumonia. Why, therefore, should any considerable amount of fluid, endangering a necessary vital structure in these respects, be allowed to accumulate under the idea of the danger of interference, which could not possibly be greater than the dangers of non-interference ? If, then, there is an effusion evidently not inclined to disperse, causing at the same time venous congestion, difficult respiration or cyanosis, the teachings of all surgical interference for like effusions into the abdominal cavity point to direct abstraction, without delay or blind trust in the powers of nature, which, while they may seem to be equal to the emergency, are as often overtaxed and thus give place for subsequent and more serious lung disease.

But if this reasoning is true in simple effusions, it is all the more to be adopted in empyema. Indeed, the lines between the two forms of the disease are often so illy determined, that in doubtful cases, if for no other reason, aspiration is necessary to determine the nature of the fluid. More than a few cases of effusion are considered simple, until aspiration reveals the fact that they are purulent. Early puncture in simple cases would be justified from this standpoint alone, were there no other advantages attending it.

Toussaint's cases show the value of early aspiration in pleurisy:

| | | | | | | | | | | | |
|---|---|---|---|---|---|---|---|---|---|---|---|
| 4 | deaths | in | 176 | cases | operated | on | between | 1st | and | 20th | day. |
| 6 | " | " | 80 | " | " | " | " | 20th | " | 60th | " |
| 1 | " | " | 7 | " | " | " | " | 60th | " | 120th | " |

With such a showing as this, the folly of waiting for a late date to do an operation which is in itself simple and safe, but which grows more dangerous as it is delayed, becomes more apparent. A procedure which is efficient at an early day is of more than doubtful value when deferred. It is said that hope deferred maketh the heart sick, but here an operation from which primarily much is to be hoped, later on can promise nothing unless failure, which is not attributable to the operation, but to the conditions which are met when it is performed.

The record of delay in the treatment of suppurative pleuritis is one burdened with complications and mortality. The value of early puncture, if in suspicious cases only, needs no urging, but from the single fact that empyema may exist and give no signs, apart from that of ordinary effusion.

Indeed, remedies may seem to control the effusion and tonics to benefit the patient, when suddenly there is exacerbation of the symptoms, followed by collapse and death in a short time. The lesion in these cases has resulted in gangrene of the lung, either from pressure or necrotic changes from simple purulent irritation, or from deficient nutrition of any origin whatever. With such complications as these, are we to wait for heart involvement, or pulmonary edema in the remaining sound lung tissue, before prompt surgical interference is resorted to ?

The lamentable failures of medicaments here would be ludicrous if not so disastrous. Pot. iodide and tr. of digitalis are urged and with stimulants and tonics, only to give a spurious strength and a delusive hope.

The heart is oppressed without by the effusion and within by the digitalis, until, broken by its double labors, it grows weaker, while the gasping, cyanotic patient lives, an enigma unsolved and unsuccored by an ignorance as profound as its faith is wild. This is a strong picture, but not exaggerated. While the text-books dwell only upon the expediency of aspiration in empyema, they are stopping short of the limit wherein safety lies. The performance is not to be regarded as a simple expedient, but as a *necessity*. There is nothing before it — nothing short of it — nothing but it. If there is doubt, so much the more is it to be urged; for the doubt is removed, and by it one of two fluids, either of which is harmful, differing only in degree.

A pint of fluid removed, clearly and entirely, is far better outside the thorax, be it pus or serum.

This is a direct teaching of abdominal surgery, whose truth is daily confirmed. Pus anywhere within the body is less dangerous by reason of an operation to remove it.

Aspiration, if insufficient by one puncture, is to be replaced by subsequent more radical procedure. By this we come to the question, what shall the treatment be? Various procedures have been urged — simple drainage, double puncture with drainage, trephining and resection.

In exceptional cases an extensive resection may be necessary, just as in simple cases the simplest form of drainage may be efficient. I incline to the belief that as an average procedure, readily to be accomplished by the general practitioner with average surgical knowledge, trephining will be the most satisfactory operation, both for results and facility of performance, for the following reasons: 1st. If two openings are made into the thorax, the entrances will be large enough easily to admit a drainage-tube of sufficient caliber to wash out the thorax. 2d. Generally these openings will be large enough to give time for cicatrization — granulation of the empyemic membrane before they close. 3d. The openings, while large enough for cleansing purposes, are not so large as to give trouble in dressing, such as must be present, to a greater or less extent, when an extensive resection is made.

With such an opening, a drainage-tube is easily introduced and kept clean, simple antiseptic precaution, without elaboration, being all that is required. I am strongly of the opinion that in these operations the use of corrosive antiseptics, for the prevention of putridity or acrid and offensive discharges, is more necessary than in abdominal operations.

So far as washes (intra-thoracic) are concerned, I doubt much whether they are to be urged as necessary, save in offensive discharges. The exception to this is iodine, which seems well deserving of credit for an influence in hastening a healthy, or a non-suppurative tendency in the pleura.

The washings are to be more regarded as an efficient means of keeping the drainage-tube and passage clean than as having any curative effect upon the tissues, and are never harmful if of sufficiently high temperature to prevent shock. The surgical details of the operation need not be here considered.

126 West Diamond Street.

